# Impact of cell shape in hierarchically structured plant surfaces on the attachment of male Colorado potato beetles (*Leptinotarsa decemlineata*)

**DOI:** 10.3762/bjnano.3.7

**Published:** 2012-01-23

**Authors:** Bettina Prüm, Robin Seidel, Holger Florian Bohn, Thomas Speck

**Affiliations:** 1 Plant Biomechanics Group Freiburg, Botanic Garden, Faculty of Biology, University of Freiburg, Schänzlestraße 1, 79104 Freiburg, Germany; 2 Bionics Competence Network BIOKON e. V., Ackerstraße 76, 13355 Berlin, Germany; 3 Competence Network Biomimetics, Schänzlestraße 1, 79104 Freiburg, Germany

**Keywords:** cuticular folds, epicuticular wax crystals, insect–plant interaction, papillae, structure–function relationship

## Abstract

Plant surfaces showing hierarchical structuring are frequently found in plant organs such as leaves, petals, fruits and stems. In our study we focus on the level of cell shape and on the level of superimposed microstructuring, leading to hierarchical surfaces if both levels are present. While it has been shown that epicuticular wax crystals and cuticular folds strongly reduce insect attachment, and that smooth papillate epidermal cells in petals improve the grip of pollinators, the impact of hierarchical surface structuring of plant surfaces possessing convex or papillate cells on insect attachment remains unclear. We performed traction experiments with male Colorado potato beetles on nine different plant surfaces with different structures. The selected plant surfaces showed epidermal cells with either tabular, convex or papillate cell shape, covered either with flat films of wax, epicuticular wax crystals or with cuticular folds. On surfaces possessing either superimposed wax crystals or cuticular folds we found traction forces to be almost one order of magnitude lower than on surfaces covered only with flat films of wax. Independent of superimposed microstructures we found that convex and papillate epidermal cell shapes slightly enhance the attachment ability of the beetles. Thus, in plant surfaces, cell shape and superimposed microstructuring yield contrary effects on the attachment of the Colorado potato beetle, with convex or papillate cells enhancing attachment and both wax crystals or cuticular folds reducing attachment. However, the overall magnitude of traction force mainly depends on the presence or absence of superimposed microstructuring.

## Introduction

In plants the cuticle constitutes the outermost layer of the plant body and provides the direct interface to the environment. The cuticle is known to show multifaceted surface structuring and to serve different functions. Besides stabilisation of the plant tissue and reduction of uncontrolled water loss by providing a transport barrier, the cuticle, e.g., influences surface wetting and sometimes allows for self-cleaning by draining of water. Furthermore, the cuticle can provide protection against harmful radiation, influences the optical properties of the plant surface, and can either improve or impede attachment of insects [1–2].

Structuring of epidermal surfaces such as leaves, petals and stems is manifold and occurs on different levels, leading to hierarchical organisation [3]. Both the shape and orientation of surface structuring have been described and named by Barthlott and co-workers [4–5] and in the review of Koch et al. [1]. In the present study we focus on the cellular level (shape of epidermal cells) and on the level of superimposed microstructuring, such as wax crystals (wc), cuticular folds (cf), or only films of wax (o).

On the cellular level, besides tabular cells (i), convex epidermal cells (ii) are frequently found on plant surfaces and are the most common cell type in epidermal surfaces [1]. Another type of cells is papillate epidermal cells (iii), which are found in particular on the surfaces of angiosperm petals [2]. The function of papillate epidermal cells in petals has been investigated in several studies in the recent years (reviewed in [2]) and the cellular structure was reported to influence the colour and wetting properties of the flower and to improve the grip of pollinating insects [6]. At the hierarchical level of superimposed microstructuring, both wax crystals and cuticular folds have been shown to influence insect attachment strongly [7–8] and also the wettability of the surface [9–10].

Many plant surfaces possess hierarchical structuring but only a few of them have been analysed with regard to their function. Leaves of *Nelumbo nucifera* (lotus) and *Colocasia esculenta* for example show papillate epidermal cells covered with epicuticular wax crystals [9], while petals of *Viola tricolor* have papillate epidermal cells covered with cuticular folds [10]. All three surfaces have been shown to be superhydrophobic and anti-adhesive for water. Other petals showing papillate cells covered with cuticular folds, such as petals of roses, were hydrophobic but of high adhesion for water [10–11]. Hierarchical structuring of different characteristics has also been found in carnivorous plants and kettle trap flowers. Plant species from different systematic groups were investigated and classified as to their surface design by Poppinga et al*.* [12]. Surface structuring, showing anisotropic papillate cells pointing downwards are frequently found in carnivorous plants and kettle trap flowers and have been proposed to increase slipperiness [12]. In some kettle trap flowers, as well as on many leaves and petals, noninclined convex or papillate cells covered with either wax crystals or cuticular folds are found. However, their impact on the attachment ability of insects remains unclear and has to our knowledge not been quantitatively investigated so far. In the present study we focus on the question of by how much a hierarchically organised surface is able to reduce attachment forces and whether cell shape influences the attachment ability of insects in hierarchical plant surfaces covered with epicuticular wax crystals or cuticular folds.

## Results

### Morphology

**Figure 1:**
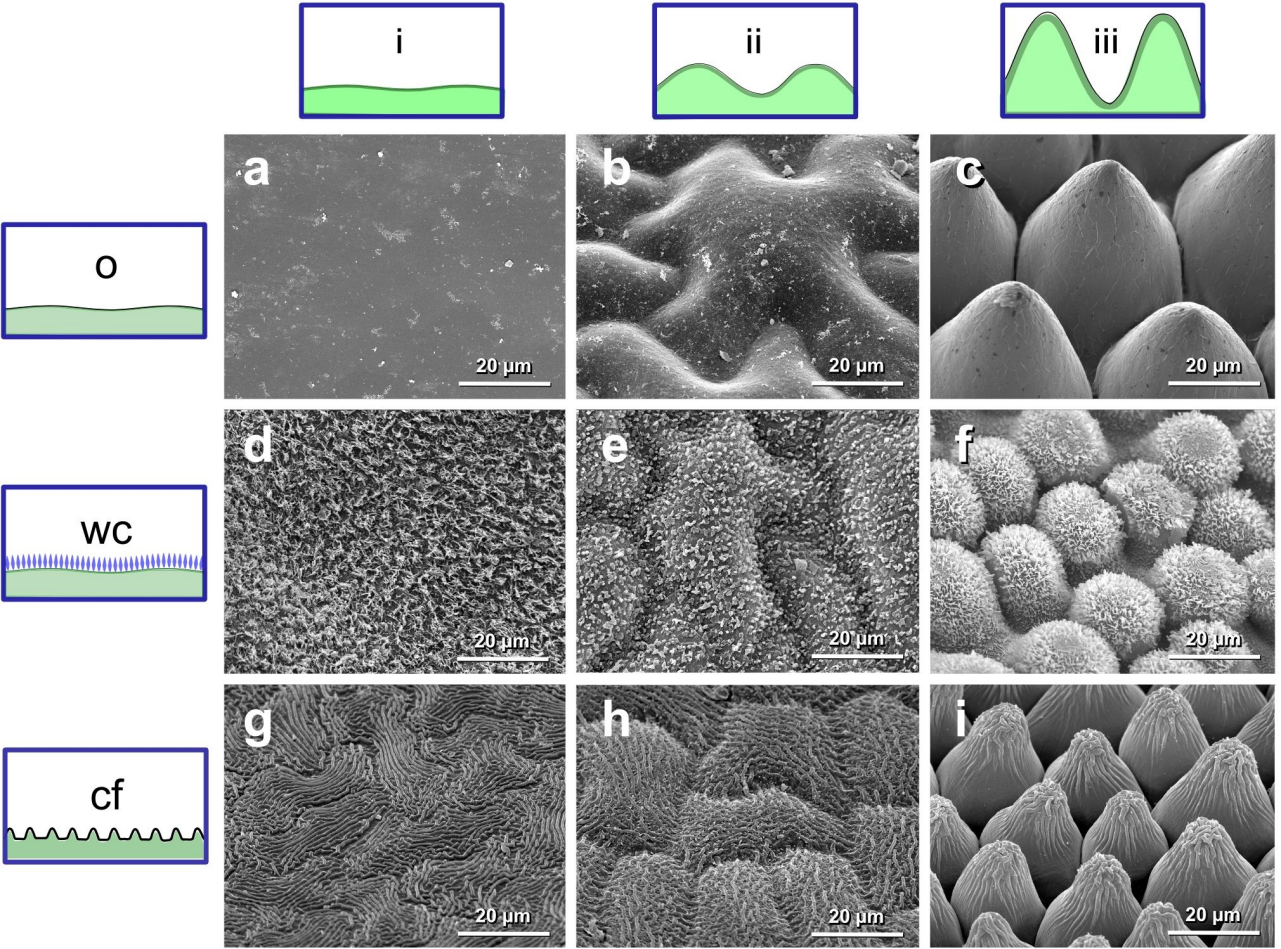
Plant surfaces investigated showing different types of structuring. Pictograms on the top show the shape of the epidermal cells: Tabular cells (i), convex cells (ii) and papillate cells (iii). Pictograms on the left illustrate the level of superimposed microstructuring: Films of wax / no further structuring (o), epicuticular wax crystals (wc) and cuticular folds (cf). SEM micrographs of plant surfaces: (a) *Magnolia grandiflora*, (b) *Paeonia officinalis*, (c) *Calathea zebrina*, (d) *Diospyros kaki*, (e) *Paeonia suffruticosa*, (f) *Colocasia esculenta*, (g) *Hevea brasiliensis*, (h) *Vitis vinifera*, (i) *Rosa* hybrid Floribunda cv*.* “Sarabande”.

Plant surfaces were classified according to their structuring both on the cellular level and on the level of superimposed microstructuring (Figure 1). Dimensions of both diameter and height of the curvature of epidermal cells are summarised in Table 1.

**Table 1:** Dimensions of structuring of plant surfaces tested (*n* = 10). Aspect ratio β = width/height of epidermal cell curvature. n/a = not applicable.

type of structuring	plant species	Epidermal cells				Wax crystals			Cuticular folds	
				
		diameter (µm)	height (µm)	aspect ratio β		width (µm)	spacing (µm)		width (µm)	spacing (µm)

i+o	*M. grandiflora*	n/a	n/a	n/a		n/a	n/a		n/a	n/a
ii+o	*P. officinalis*	52.1 ± 28.3	8.2 ± 1.8	6.3		n/a	n/a		n/a	n/a
iii+o	*C. zebrina*	38.7 ± 4.7	52.9 ± 8.6	0.7		n/a	n/a		n/a	n/a

i+wc	*D. kaki*	n/a	n/a	n/a		<0.1	0.6 ± 0.6		n/a	n/a
ii+wc	*P. suffruticosa*	46.4 ± 21.6	6.6 ± 2.1	7.0		~0.1	0.6 ± 0.4		n/a	n/a
iii+wc	*C. esculenta*	19.4 ± 3.7	16.2 ± 1.1	1.2		<0.1	0.3 ± 0.1		n/a	n/a

i+cf	*H. brasiliensis*	n/a	n/a	n/a		n/a	n/a		0.5 ± 0.1	0.8 ± 0.5
ii+cf	*V. vinifera*	22.2 ± 6.7	4.5 ± 0.6	4.9		n/a	n/a		0.6 ± 0.1	1.2 ± 0.5
iii+cf	*Rosa* hybrid	20.9 ± 3.5	25.2 ± 2.5	0.8		n/a	n/a		0.7 ± 0.1	0.6 ± 0.5

Focusing on the level of superimposed microstructuring, we selected plant surfaces only covered with films of wax (*Magnolia grandiflora* (i+o), *Paeonia officinalis* (ii+o) and *Calathea zebrina* (iii+o)), plant surfaces covered with a dense layer of epicuticular wax crystals (*Diospyros kaki* (i+wc), *Paeonia suffruticosa* (ii+wc) and *Colocasia esculenta* (iii+wc)), and plant surfaces showing cuticular folds similar in size (*Hevea brasiliensis* (i+cf), *Vitis vinifera* (ii+cf) and *Rosa* hybrid Floribunda cv. “Sarabande” (iii+cf)). For dimensions of the superimposed microstructures of the surfaces investigated, see Table 1.

### Traction experiments

**Figure 2:**
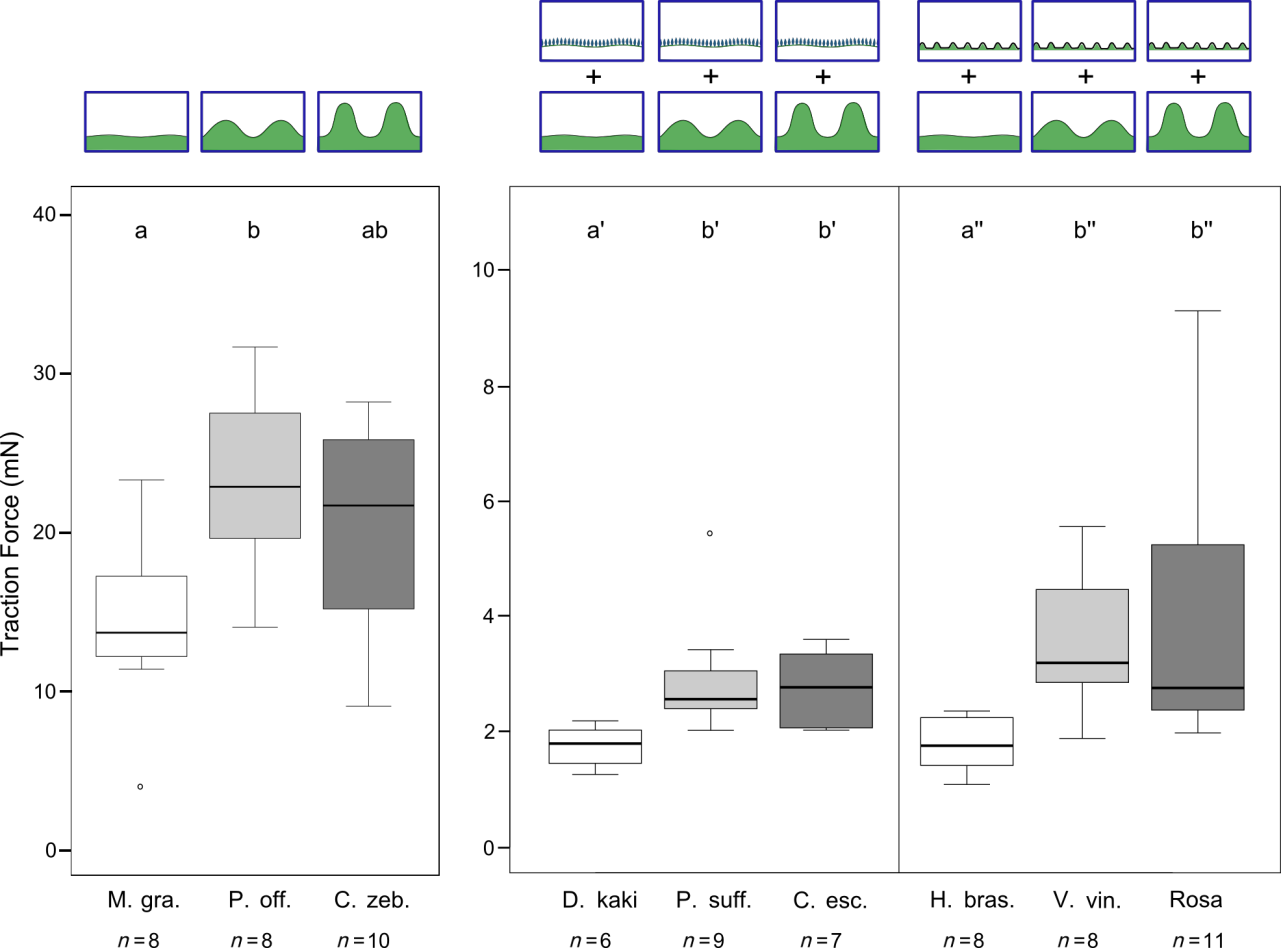
Traction forces of actively walking male *Leptinotarsa decemlineata* beetles on plant surfaces of different structure, both on the cellular level and on the level of superimposed microstructuring. Significant differences in traction force between tabular, convex and papillate epidermal cells are indicated by lower case letters. M. gra: *Magnolia grandiflora;* P. off: *Paeonia officinalis;* C. zeb.: *Calathea zebrina,* D. kaki: *Diospyros kaki,* P. suff.: *Paeonia suffruticosa* , C. esc.: *Colocasia esculenta*, H. bras.: *Hevea brasiliensis*, V. vin.: *Vitis vinifera*, Rosa: *Rosa* hybrid Floribunda cv. “Sarabande”.

By comparison of traction forces on plant surfaces of different cell shape (Figure 2), significant differences between the groups i, ii and iii were found for all types of microstructuring (Kruskal–Wallis one way ANOVA on ranks. i+o, ii+o, iii+o: *p* = 0.023; i+wc, ii+wc, iii+wc: *p* = 0.01; i+cf, ii+cf, iii+cf: *p* = 0.001). Although significant differences were found between plant surfaces with different cell shapes, traction forces on surfaces showing wax crystals or cuticular folds were almost an order of magnitude lower compared to plant surfaces covered only with films of wax.

For plant surfaces covered only with films of wax, there was a significant increase in traction force when comparing convex to tabular epidermal cells (i+o (*n* = 8), ii+o (*n* = 8), *p* = 0.03). Between surfaces showing convex and papillate cells no further increase in traction force was found (ii+o (*n* = 8), iii+o (*n* = 10), *p* = 0.564). Comparing traction forces on tabular to papillate cells, the differences between the groups were also found to be insignificant (i+o (*n* = 8), iii+o (*n* = 10), *p* = 0.091).

On plant surfaces showing hierarchical organisation by a superimposed structure, either by wax crystals or by cuticular folds, the differences in traction force between cells of different shape were more pronounced, although significantly lower in absolute value than for plant surfaces covered only with films of wax. For plant surfaces covered with wax crystals, there was a significant increase in traction force when comparing convex to tabular epidermal cells (i+wc (*n* = 6), ii+wc (*n* = 9), *p* = 0.017). Between surfaces showing convex and papillate cells no further increase in traction force was found (ii+wc (*n* = 9), iii+wc (*n* = 7), *p* = 1). Comparing traction forces on tabular to papillate cells, the differences between the groups were significant (i+wc (*n* = 6), iii+wc (*n* = 7), *p* = 0.037). For plant surfaces showing cuticular folds, there was a highly significant increase in traction force when comparing convex to tabular epidermal cells (i+cf (*n* = 8), ii+cf (*n* = 8), *p* = 0.006). Between surfaces showing convex and papillate cells there was no significant increase in traction force (ii+cf (*n* = 8), iii+cf (*n* = 11), *p* = 0.65). When comparing traction forces between tabular and papillate cells, the differences were found to be highly significant (i+cf (*n* = 8), iii+cf (*n* = 11), *p* = 0.004).

Despite the significant differences between surfaces of different cell shape, the absolute values of traction forces on all plant surfaces covered either with wax crystals or cuticular folds were significantly lower than on surfaces covered only with films of wax.

## Discussion

**Figure 3:**
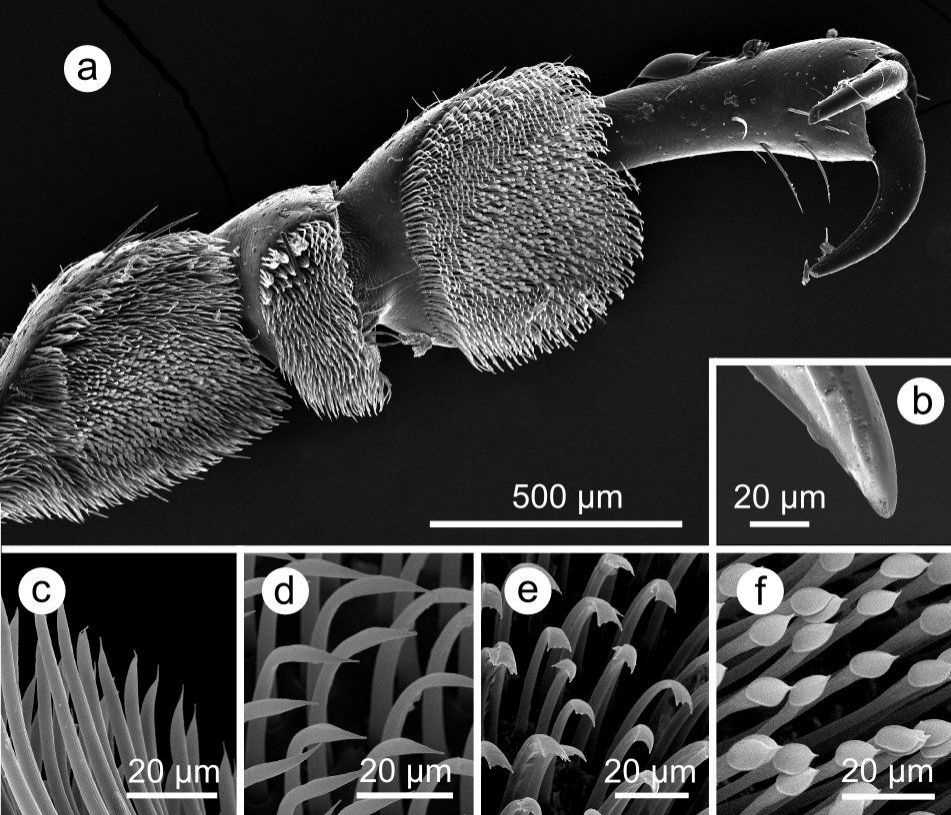
SEM micrographs of the attachment devices in a male *Leptinotarsa decemlineata*. (a) Ventral view of a hind leg; (b) claw tip; (c–f): Tarsal adhesive setae: (c) filamentous, (d) lanceolate, (e) spatula-shaped and (f) discoidal setae. Terminology according to Voigt et al. [13]. Adapted from [8].

Our results show that, independent of cell shape, an additional hierarchical level of superimposed microstructuring leads to a strong reduction of traction forces. In comparison to the surfaces where no additional level of hierarchy was present, the traction forces are reduced in similar amounts for wax crystals and cuticular folds. For attachment of *L. decemlineata*, as for many other insects, both adhesive pads and claws are responsible (Figure 3). The hairy adhesive pads of beetles show best attachment on smooth surfaces, or on surfaces with very large diameters of the asperities, as shown in experiments with insects having dissected claws [13–17]. For various insects it has been shown that roughness induced by wax crystals [7,18–23] or cuticular folds [8] strongly reduces attachment ability. Different mechanisms have been hypothesised [20,24], but the roughness hypothesis is most frequently put forward, in which is it proposed that a reduction in the real contact area between the attachment device and the substrate is responsible for a reduction of the attachment ability [7–8,13,15,17–23].

For smooth plant surfaces composed of tabular cells covered only with films of wax, traction forces were eight times higher than on smooth surfaces showing superimposed microstructuring (relation of i+o to i+wc and i+cf). For surfaces covered with films of wax, we propose that the adhesive pads of *L. decemlineata* adapt to the surface with a clearly increased contact area between the attachment device and the substrate compared to plant surfaces with superimposed microstructuring. Focusing on the cell shape, for surfaces covered with films of wax we found traction forces on convex epidermal cells to be 1.7 times the traction forces on tabular cells. However, no further significant increase in traction forces between convex and papillate epidermal cells was detected. The (macro-) roughness induced by the cell shape should provide sufficient area with smooth surface to establish a close contact between adhesive pads and the substrate [13].

On surfaces showing asperities, claws generally improve grip [14–15] depending on the dimensions of the surface asperities and the insect’s claws [15–16]. If the diameter of the claw tip is smaller than the surface roughness, the claws can hook into the surface irregularities and the beetle thereby increases attachment forces. As in *L. decemlineata* the diameter of the claw tips is 8–10 µm [8], the claws presumably are not able to interlock with wax crystals or cuticular folds, as spacing between the single structures is below 1.5 µm and thereby much too small for the claws to get into the gaps. On the other hand, diameters of convex and papillate epidermal cells are between 20 and 50 µm and hence spacing between the cells should allow the claws to interlock, while for the tested plant surfaces with tabular epidermal cells no such surface structures are given. We propose that the cell shape dependent differences in traction forces are due to an improved grip of the beetles’ claws caused by the elevated cell shape. Furthermore, friction might be increased with the setae possibly getting caught behind the elevated epidermal cells.

In plant surfaces possessing epicuticular wax crystals or cuticular folds the influence of cell shape on insect attachment was similar: Traction forces on surfaces with convex cells were 1.4 (wc) and 1.8 (cf) times as high as on surfaces possessing tabular epidermal cells with the respective microstructuring. A further elevation of cell curvature, leading to papillate cells, did not further increase traction forces. We hypothesise that the claws interlock with the cell curvature, but because of only a marginal increase in traction forces in absolute values between tabular and convex epidermal cells our data indicate that the magnitude of the traction forces is mainly due to the adhesive pads of the beetle. Similar to the findings of Voigt et al. [13], our results support the principal role of adhesive pads in attachment to both smooth and rough substrates.

On petals of *Rosa* hybrid Floribunda cv*.* “Sarabande” traction-force measurements showed a distinctively higher variation towards higher forces. Due to the softness of the petals, increased traction forces might be associated with a possible punching of the surface due to the forces applied by the claws [21]. The influence of the physicochemical properties of surfaces on insect attachment has been discussed in several studies [21,23,25–26]. Hence, differences in the surface chemistry of the plant species we tested may have an additional impact on the traction forces. Attachment of the insect involves frictional and adhesive forces, and due to the pulling direction of the insects we propose that frictional forces predominate. To understand fully the underlying mechanism of force reduction including the proportion of friction and adhesion on insect attachment, further investigations are needed.

Taking into account the multifunctionality of the plant surfaces [1], our results indicate that the main function of convex and papillate epidermal cells in hierarchically structured plant surfaces is not associated with the attachment of leaf beetles. Rather the additional level of hierarchy added by the cellular shape is likely to serve other main functions: Papillate epidermal cells covered with epicuticular wax crystals or cuticular folds have been shown to minimise wettability [9–10]; conical epidermal cells in petal surfaces have been reported to aid the pollinators’ grip to increase foraging efficiency [6,27] and to have a visual effect [2]. To understand fully the impact of hierarchical surface structuring on the properties of plant surfaces, further investigations are needed.

## Conclusion

We found traction forces on surfaces with either superimposed wax crystals or cuticular folds to be in the same range and almost one order of magnitude lower than on surfaces covered with only flat films of wax. Independent of the superimposed microstructure, the attachment ability of the beetles on convex and papillate epidermal cell shapes was slightly enhanced. We assume that this increase of attachment forces is mainly caused by an interlocking of the beetles’ claws with the curvature in convex and papillate cells. The cell shape and superimposed microstructuring have opposite effects on the attachment of the Colorado potato beetle, with convex and papillate cells enhancing and both wax crystals or cuticular folds reducing attachment. Our data show that the magnitude of the traction force is determined mainly by the existence of superimposed microstructuring. Thus the influence of cell shape on the attachment of the beetle is much lower than the influence of the superimposed microstructuring. These results contribute to a better understanding of the functions of multifaceted surface structuring as a basis for the development of biomimetic technical surfaces.

## Experimental

### Insects and plant species

Beetles constitute the largest order within the class of insects, and their attachment pads are of the hairy type [28]. In the present study the Colorado potato beetle *Leptinotarsa decemlineata* (Coleoptera: Chrysomelidae) was used as a model insect species. This leaf beetle has frequently been used as model insect species for traction experiments and its attachment devices have been well analysed [8,13,29–30]. The tarsus (Figure 3) consists of five tarsomeres, with tarsomeres 1–3 being covered with setae of four different types [13], the fourth tarsomere being hidden, and a pair of curved claws on the pretarsus [13]. The diameter of the claw tips is 8–10 µm [8]. Beetles were partly collected at organic potato fields in the Kaiserstuhl area near Freiburg and partly obtained from the Julius Kühn Institut in Darmstadt and kept in a terrarium on their host plant, *Solanum tuberosum*, using a day–night regime of 16L:8D (Osram Lumilux Daylight 865 lamp, 58 W). For experiments only male beetles were used, which were 11–14 mm in length and with a body mass ranging from 90 to 140 mg [8].

To investigate the influence of hierarchical structuring on insect attachment, nine plant surfaces (Table 2) showing different types of hierarchical structuring were chosen. On the level of cell shape we focused on plant surfaces with (i) tabular, (ii) convex and (iii) papillate epidermal cells, termed according to the aspect ratio, width to height, of the cell curvature (aspect ratio β = width/height. convex: 10 > β > 3; papillae: 1.5 > β > 0.5; the height of cell curvature in tabular cells is close to zero and therefore this description cannot be applied) modified after Barthlott [4] and reviewed by Ehler [1]. On the level of superimposed microstructuring we analysed plant surfaces showing (o) only flat films of wax (without any further structuring), (wc) epicuticular wax crystals and (cf) cuticular folds. All plants used were grown in the Botanical Garden of the University of Freiburg, except for *Calathea zebrina*, which was obtained from a commercial garden shop.

**Table 2:** Plant species selected. In square brackets: Accession number of the Botanical Garden of Freiburg.

		Shape of epidermal cells		

		tabular (i)	convex (ii)	papillate (iii)

Super- imposed micro- structuring	films of wax (o)	*Magnolia grandiflora* (adaxial leaf surface) [505-563]	*Paeonia officinalis* (adaxial leaf surface) [3501-383]	*Calathea zebrina* (adaxial leaf surface, dark stripes) [n/a – obtained from garden shop Sumser, Teningen]

	epicuticular wax crystals (wc)	*Diospyros kaki* (fruit surface) [900-87]	*Paeonia suffruticosa* (adaxial leaf surface) [3501-385]	*Colocasia esculenta* (adaxial leaf surface) [3801-652]

	cuticular folds (cf)	*Hevea brasiliensis* (adaxial leaf surface) [old accession]	*Vitis vinifera* ssp. *sylvestris* (abaxial leaf surface) [1000-1]	*Rosa* hybrid Floribunda (cultivar “Sarabande”) (petal, adaxial surface) [3402-0]

### Morphology

The morphology of the plant surfaces was characterised by scanning electron microscopy (SEM). For investigation of epicuticular wax crystals, the samples were mounted on aluminium stubs without any prior dehydration step. For SEM analysis of cell shape and cuticular folds, leaf samples were dehydrated in methanol [31] and critical point dried (LPD 030, Bal-Tec). For cross-section analysis of plant surfaces, the critical-point-dried samples were cut with a razor blade. Samples were mounted on aluminium stubs by means of conductive double-sided adhesive tabs (Plano, Wetzlar, Germany). All samples were sputter coated with gold (approximately 15 nm; cressington sputter coater, 108 auto) and examined in a Leo 435 vp SEM (Leica, Wiesbaden, Germany). Dimensions of cell shape and superimposed microstructuring were estimated by examining the cross-section and top view SEM-images by using the software ImageJ (v. 1.44p, National Institutes of Health, USA; http://rsb.info.nih.gov/ij/). Each type of structuring was measured at ten different positions (*n* = 10). As epidermal cells can differ greatly in diameter between different species [2], we selected plant species having convex and papillate cells of the same order of magnitude. Shapes of epidermal cells were termed after the classification of Barthlott [4], reviewed by Ehler [1], and the shape of epicuticular wax crystals after Barthlott et al. [5].

### Traction experiments

**Figure 4:**
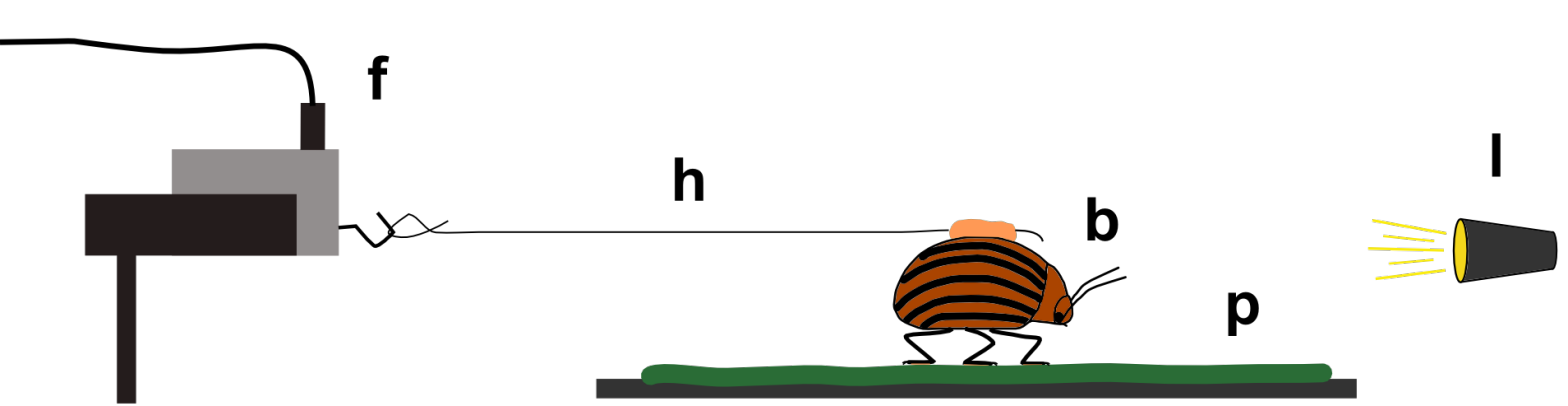
Experimental setup. Traction forces of a beetle (b) actively walking on a plant surface (p) were recorded by using a highly sensitive force transducer (f). The beetle was fixed to the force transducer by a piece of hair (h) and attracted by a small light source (l). Adapted from [8].

Traction experiments were performed as described in detail by Prüm et al. [8]. For traction experiments, plant samples were fixed to a glass slide by means of double-sided adhesive tape and the test substrate was subsequently aligned horizontally on the stage. Maximum traction forces of actively walking beetles were investigated by tethering individual beetles to a highly sensitive force transducer (Fort 25, World Precision Instruments Inc., Sarasota, USA), by using a human hair attached to the beetle’s elytra (Figure 4). The force was recorded during at least 2 min of active walking on the respective test substrate, and the measurement was stopped if the beetle did not walk straight in the forwards direction. Within each measurement the median of the 15 highest local maxima with a minimum time lag of three seconds between two neighbouring peaks was extracted. Measurements were repeated with 6–11 different beetles per plant species. For each run a fresh piece of leaf was used.

### Statistics

Data were analysed by using Kruskal–Wallis one way ANOVA on ranks tests followed by Mann–Whitney–Wilcoxon tests for pairwise comparison of means. Due to multiple testing, *p*-values were corrected according the “Holm” method. All statistical tests were performed by using R (v. R 2.9.1).
